# Is probabilistic cuing of visual search an inflexible attentional habit? A meta-analytic review

**DOI:** 10.3758/s13423-021-02025-5

**Published:** 2021-11-23

**Authors:** Tamara Giménez-Fernández, David Luque, David R. Shanks, Miguel A. Vadillo

**Affiliations:** 1 Departmento de Psicología Básica, Universidad Autónoma de Madrid, Madrid, Spain; 2 Departmento de Psicología Básica, Universidad de Málaga, Malaga, Spain; 3 Division of Psychology and Language Sciences, University College London, London, United Kingdom

**Keywords:** Habitual attention, Implicit learning, Meta-analysis, Probabilistic cuing, Visual search

## Abstract

﻿In studies on probabilistic cuing of visual search, participants search for a target among several distractors and report some feature of the target. In a *biased stage* the target appears more frequently in one specific area of the search display. Eventually, participants become faster at finding the target in that rich region compared to the sparse region. In some experiments, this stage is followed by an *unbiased stage*, where the target is evenly located across all regions of the display. Despite this change in the spatial distribution of targets, search speed usually remains faster when the target is located in the previously rich region. The persistence of the bias even when it is no longer advantageous has been taken as evidence that this phenomenon is an attentional habit. The aim of this meta-analysis was to test whether the magnitude of probabilistic cuing decreases from the biased to the unbiased stage. A meta-analysis of 42 studies confirmed that probabilistic cuing during the unbiased stage was roughly half the size of cuing during the biased stage, and this decrease persisted even after correcting for publication bias. Thus, the evidence supporting the claim that probabilistic cuing is an attentional habit might not be as compelling as previously thought.

**Supplementary Information:**

The online version contains supplementary material available at 10.3758/s13423-021-02025-5.

## Introduction

The allocation of attentional resources to objects in the environment is influenced by previous experience (Gaspelin & Luck, 2018; Theeuwes, 2018; Vecera et al., 2014). One way in which previous experience shapes attention is by inducing a bias toward the probable location of the item we are searching for. For example, when we want to turn on the lights in a room, we look for the switch at locations around half the height of the door, next to it and inside the room, because based on our previous experience we know that this is where switches usually are. This phenomenon, known as probabilistic cuing or location probability learning, improves the efficiency of visual search (e.g., Geng & Behrmann, 2005). In a typical experiment exploring this form of attentional bias (e.g., Druker & Anderson, 2010), participants search for a visual target among several distractors and report some feature of the target, such as its identity. Unknown to participants, the target is more frequently located in one specific area of the search display (i.e., the rich region) than in the remaining areas of the display (i.e., the sparse region). As the task progresses, participants become faster at finding the target in the rich compared to the sparse region.

**Fig. 1:**
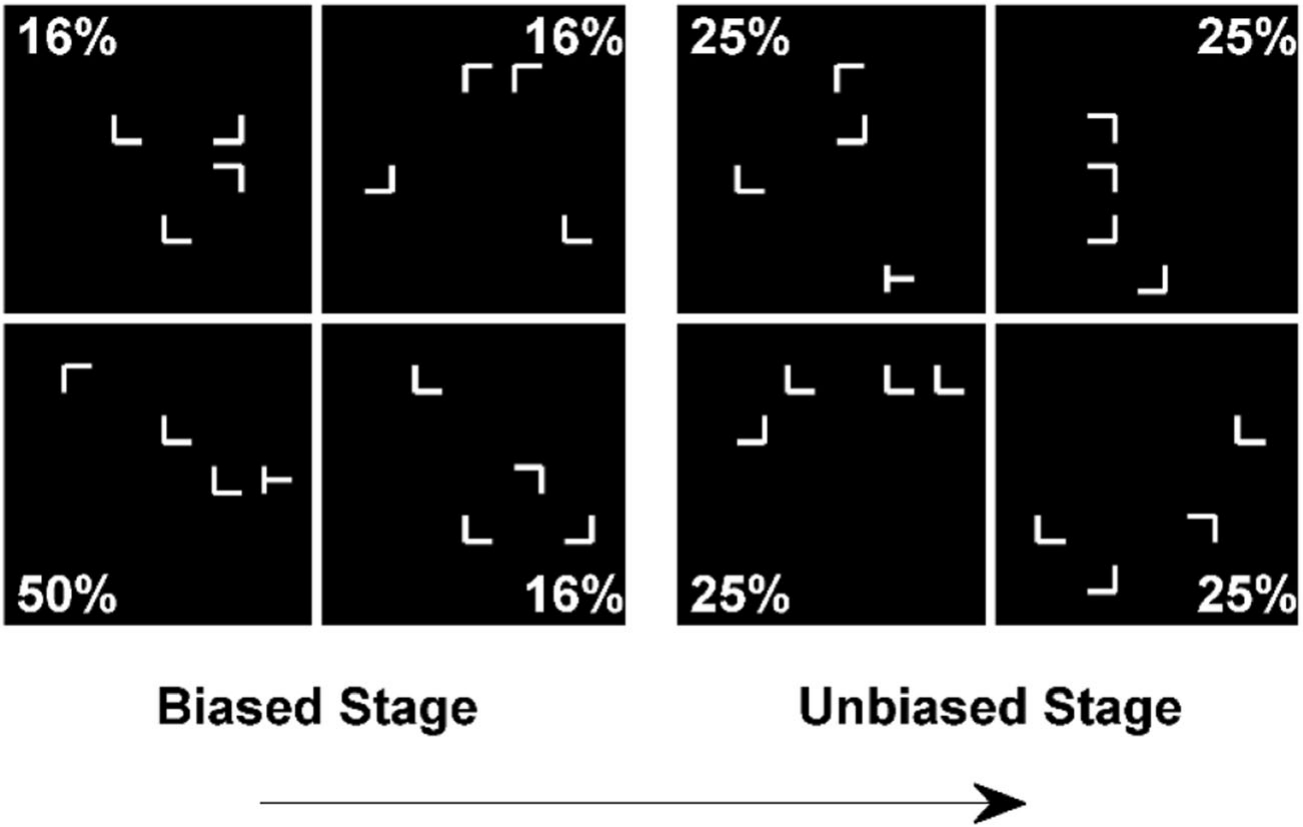
Schematic illustration of the biased and unbiased stages of a probabilistic cuing task. In both stages the participant’s task is to report, pressing a key, the left or right orientation of the tilted T. In the biased stage the T appears in one of the quadrants of the display (lower left in this example) on half of the trials. In the unbiased stage, it appears evenly distributed across quadrants

Some authors have claimed that this phenomenon may be due to repetition priming. It is often easier to find a visual target if it appears in the same location as in the previous trial. In probabilistic cuing experiments, these repetitions are more likely to take place in the rich region, because that is the area of the display where the target appears most frequently. This, on its own, could explain why search times are faster in this region (Walthew & Gilchrist, 2006). To minimize the possibility that probabilistic cuing is driven by fleeting inter-trial priming processes, most studies include two stages (e.g., Addleman et al., 2019; Hong et al., 2020). In the *biased* learning stage, participants carry out the probabilistic cuing task as described above for several hundreds of trials. This stage is followed by an *unbiased* testing stage, where the target is evenly located across the different regions of the display. Figure 1 shows a schematic illustration of the displays and design in a typical experiment. Despite this change in the spatial distribution of targets, participants usually continue to respond faster in the unbiased stage when the target is located in the previously rich quadrant (e.g., Ferrante et al., 2018; Lee et al., 2020). This result can no longer be explained in terms of repetition priming, because during the unbiased stage repetitions are equally likely to appear in all regions.

Once repetition priming was discounted as the explanation for the probabilistic cuing effect (Jiang, Swallow, Rosenbaum, & Herzig, 2013c), it was proposed that it was better understood as an enduring attentional habit (Jiang & Sisk, 2019). An attentional habit is conceptualized as being “gradually learned, occurring repeatedly and remarkably fixed, automatic and unconscious, and typically involving a structured action sequence elicited by particular contexts” (Jiang & Sisk, 2019, p. 65). This interpretation was supported not only by the evidence that the bias persisted during the unbiased stage but also by the fact that it did not decline at all (Addleman et al., 2019, Experiment 1; Jiang et al., 2015a, 2016; Jiang & Swallow 2013a, 2013b; Jiang, Swallow, & Sun, 2014a; Jiang, Swallow, Rosenbaum, & Herzig, 2013c; Sisk et al., 2018). This result resembles habitual processes as studied in other areas of research, where habit learning seems to be slow and highly persistent (Bayley et al., 2005; Dickinson, 1985).

However, the evidence supporting the inflexibility of probabilistic cuing is nuanced. Other studies have consistently found evidence of a decrease of the size of probabilistic cuing during the unbiased stage (Addleman et al., 2021, Experiment 1; Giménez-Fernández et al., 2020; Jiang, Won, & Swallow, 2014b; Sha et al., 2018). In particular, in a high-powered (*N* = 160) previous study conducted in our laboratory (Giménez-Fernández et al., 2020) we found robust evidence of this reduction, confirming that the bias becomes attenuated as participants accumulate more and more evidence that the target is evenly distributed. We suspect that these discrepancies regarding the inflexibility of probabilistic cuing are probably due to low statistical power in some experiments. Many of the studies that have failed to find a significant decline in the attentional bias during the unbiased stage might have simply been underpowered to detect such a decrease. Of course, it is also possible that the contrast between the results observed in Giménez-Fernández et al. (2020) and those of previous studies reporting no decrease of cuing during the unbiased stage is not due to the higher statistical power of the former, but to some other unknown but crucial difference in the experimental procedure.

In sum, many studies have obtained null results regarding bias attenuation, consistent with the habit interpretation (we discuss in more detail what the concept of an attentional “habit” means in the *Discussion*). The main goal of the present study was to test whether the decrease of probabilistic cuing during the unbiased stage is an (unreliable) peculiarity of just a handful of studies or, alternatively, is a general feature of the body of evidence collected with this task that might not always reach statistical significance due to the small samples used in many of these studies. Meta-analytic methods are ideal to put these hypotheses to the test, as the conclusions reached by collating evidence from all previous studies are necessarily more reliable than the individual results from any single study. A second reason why the attentional bias might not always decrease significantly is that the unbiased testing stage is often much shorter than the biased learning stage, possibly providing little opportunity for adapting visual search to the new contingencies. The present meta-analysis also explores this issue by testing the moderating role of the number of trials in each stage.

## Method

### Inclusion and exclusion criteria

We selected studies meeting the following criteria:

1. Only studies using a probabilistic cuing task were included. By probabilistic cuing we refer to any task in which participants have to find a target that appears more frequently in one area of the display and report some characteristic of this target or whether the target is present or not.
2. The study had to include an unbiased stage (i.e., in which the target’s spatial distribution is even) after the biased stage (i.e., in which the target’s spatial distribution is biased) in which both the task and the stimuli were the same as in the biased stage.
3. The report included a contrast between the condition in which the target appeared in the rich region compared to when it appeared in the sparse region both in the biased and in the unbiased stage. If the study did not report these contrasts but it was possible to calculate them with the data collected in the experiment, we emailed the authors of the study to request this information. If 15 days later we had obtained no response, we sent a reminder. Fifteen days after that, if we still had not received a response, we excluded the study.
4. Participants had to be naïve about the spatial distribution of the target throughout the whole experiment and their attention must not have been explicitly drawn towards any region in particular.
5. We excluded studies conducted under conditions that could potentially reduce or abolish probabilistic cuing. For instance, most research has shown that the attentional bias learned in these experiments is viewer-centered. Therefore, we excluded studies in which the rich target location did not remain constant from the participants' perspective during the first stage of the experiment (Jiang & Swallow, 2014, Experiments 1A, 1B, 2 and 3; Jiang, Swallow, & Capistrano, 2013a, Experiments 1, 2 and 4). For the same reason, we also excluded studies or experimental conditions where participants performed the task under abnormal viewing conditions (e.g., a simulated scotoma; Addleman et al., 2021, Experiment 2).
6. Experiments carried out in a three-dimensional (3D) virtual reality environment or in an outdoors setting were excluded to reduce heterogeneity in experimental settings.

### Literature search strategy

The probabilistic cuing task has been given different names in the literature, and it is difficult to find a small set of search keywords that would ensure the retrieval of all relevant studies. Thus, the starting point of our literature search was a recent meta-analysis conducted by Vadillo et al. (2020) that gathered all the studies using the probabilistic cuing task that had been published until 10 November 2017. Given that our eligibility criteria were different from those of Vadillo et al. (2020), we inspected the method section of the 44 included and excluded papers in that meta-analysis. Thirteen papers were selected for inclusion. These 13 papers contained 23 studies that met the inclusion criteria explained above and which were included in the present meta-analysis.

To update Vadillo et al.’s (2020) literature search we adopted the same strategy. On 1 October 2020 we accessed the Web of Science to find new published studies on probabilistic cuing authored by Y. Jiang (the most prolific author regarding probabilistic cuing). Reading the titles and the abstracts of the 46 papers authored by this researcher between November 2017 and October 2020 we discarded 44. Two reports were further assessed for inclusion. Both were finally included in the meta-analysis. Then, we searched for additional papers inspecting their reference sections, and found one more article. These three additional papers contained six valid studies that were included.

Additionally, to retrieve unpublished data (e.g., theses or preprints), we contacted all corresponding authors of the selected studies to ask them if they had unpublished data that fitted our inclusion criteria. Also, we entered each selected article into Google Scholar and examined all the references that cited these papers. Based on the titles and abstracts of 58 papers, we discarded all of them except for six, which were further assessed and included in the meta-analysis. These six articles contained 12 additional studies that were included.

Finally, we conducted an updated search via the Web of Science in April 2021, which allowed us to identify two more articles authored by Y. Jiang. Only one of these papers contained a study meeting the inclusion criteria. We also inspected the reference list of both papers, but this did not yield any additional reports with eligible studies. Thus, 42 studies pertaining to 23 articles were included in total. These articles are marked with an asterisk in the Reference list.

### Computation of effect size

As explained above, we were interested in comparing the bias towards the rich versus sparse region in the biased and unbiased stages. Most of the selected studies report the results of one ANOVA for the biased stage and another for the unbiased stage. Usually, these ANOVAs include a factor (e.g., quadrant) coding whether the target was presented in the rich or the sparse region. Sometimes, the authors report the results of a *t*-test comparing the dependent variable (search times) in the two different regions. Since all of these entail within-subject contrasts, we computed Cohen’s *d*_z_ for each study for these comparisons, that is, the standardized mean difference from two related measures (Lakens, 2013). This effect size estimate can be calculated by dividing the *t*-value of the test comparing the rich and sparse regions by the square root of the sample size. Since *F* = *t*^2^ when the numerator degrees of freedom is 1, *d*_z_ can also be calculated from the *F*-value of a within-subjects comparison (Rosenthal, 1991). The variance of *d*_z_ for each study was estimated as

$$ {V}_i=\frac{1}{N_i}+\frac{d_i}{2{N}_i} $$

where *N*_*i*_ and *d*_*i*_ represent the sample size and the *d*_*z*_ score of each individual study (Cumming, 2012).

All included studies contained a behavioral measure of performance. When reported, we chose reaction time as the preferred measure. Accuracy data and other dependent variables were considered instead when reaction time was not available or was not a suitable measure of performance (e.g., when presentation times of the search displays were very brief).

As explained above, in some studies, the displays were presented on a monitor placed flat on a stand and participants changed their standing position from the biased to the unbiased stage. For these studies two rich quadrants can be defined, one viewer-centered and one environment-centered. The evidence available so far suggests that probabilistic cuing is viewer-centered (Jiang & Swallow, 2013b; Jiang, Swallow, & Capistrano, 2013a). Therefore, for these studies we computed the effect size comparing the performance in the viewer-centered rich and sparse quadrants.

### Coding of moderators and study characteristics

As explained in the *Introduction,* one of the methodological factors that could explain the maintenance of the bias during the unbiased stage is that in most studies, the unbiased stage includes many fewer trials than the biased stage. We hypothesized that a short unbiased stage would provide less opportunity to learn the new spatial distribution of the target and therefore update the attentional bias. Thus, we coded the length (i.e., number of trials) of both stages to explore whether this might moderate the results.

Additionally, in some studies, the experimental setting changed from the biased to the unbiased stage in one way or another (e.g., the position of the participants with respect to the display, the characteristics of the display, or the session in which each stage was carried out). It is possible that these changes in experimental settings drew participants’ attention to potential changes in the spatial distribution of the target. To control for the possibility that the results of the meta-analysis were biased by the inclusion of these studies, we coded whether the experimental settings remained unchanged across stages and we repeated the analysis including only those studies where experimental setting did not change across stages and there was no time interval between them either. The first and last author examined all studies and coded them independently. There were no disagreements in the coding of moderators.

### Meta-analytic methods

Our main aim was to test whether the magnitude of probabilistic cuing decreases from the biased to the unbiased stage. Firstly, we estimated the average effect size in the biased and in the unbiased stages, separately, fitting random-effects models with restricted maximum likelihood estimation, as implemented in the rma function of the ‘metafor’ R package (Viechtbauer, 2010). To determine whether the effect sizes in the biased and unbiased stages were significantly different, we then ran a multi-level meta-analysis collating the effect sizes from both stages, adding a random intercept at the study level with the rma.mv ‘metafor’ function. All studies included in the meta-analysis along with the data gathered for each of them are publicly available at https://osf.io/yr3mx/.

One potential problem for meta-analytic methods is that they rely on data that could be biased by the selective publication of significant findings (Carter et al., 2019). This problem is particularly concerning in the present meta-analysis because publication bias might have different effects on the results of the biased and unbiased stages. If, as seems plausible, there is more incentive to publish significant results in the biased stage than in the unbiased stage, the effects in the biased stage would almost inevitably be larger. To address this possibility, we employed several methods to detect the potential presence of publication bias and to correct for it.

First, we visually inspected the distribution of effect sizes using funnel plots. A funnel plot is a scatterplot representing the relationship between each effect size and its standard error (or other precision measure; Sterne et al., 2005, 2011). If the literature does not suffer from publication biases, one would expect effect sizes to be independent of their precision. That is, studies with many participants (i.e., high precision) should yield highly consistent results and studies with smaller samples should yield more variable results. But, in principle, the mean effect size should be roughly similar for all studies, regardless of their sample sizes. The visual representation of the relationship between effect sizes and precision should take the form of an inverted funnel, with a narrow distribution of effect sizes among the studies with the largest samples and increasingly variable effect sizes as sample sizes decrease. However, if the literature is biased against non-significant results, this plot will often reveal an asymmetrical distribution of effect sizes, because in studies with smaller samples only large effects reach statistical significance, while studies with larger samples can yield significant results even for small effect sizes. In other words, if non-significant results are less likely to appear in the published record, this will impose an artificial relationship between study precision (i.e., sample size) and effect sizes. To assess the asymmetry of the funnel plot we used Egger’s regression test (Egger et al., 1997).

There are several methods to correct for publication bias, although unfortunately there is no consensus about which of them achieves the best estimation. Following Carter et al. (2019), we applied several. Specifically, we used trim-and-fill (Duval & Tweedie, 2000), two regression-based methods (PET and PEESE; Stanley & Doucouliagos, 2014), and Vevea and Hedges’ (1995) selection model (for a detailed technical description of these methods, see Carter et al., 2019). In all cases, our goal in using these methods was to confirm that any difference between the effect sizes observed in the two stages was not driven by differences in publication bias. In other words, we expected that the numerical difference between the stages would survive any bias-correction method.

## Results

### Does probabilistic cuing decline from the biased to the unbiased stage?

**Fig. 2:**
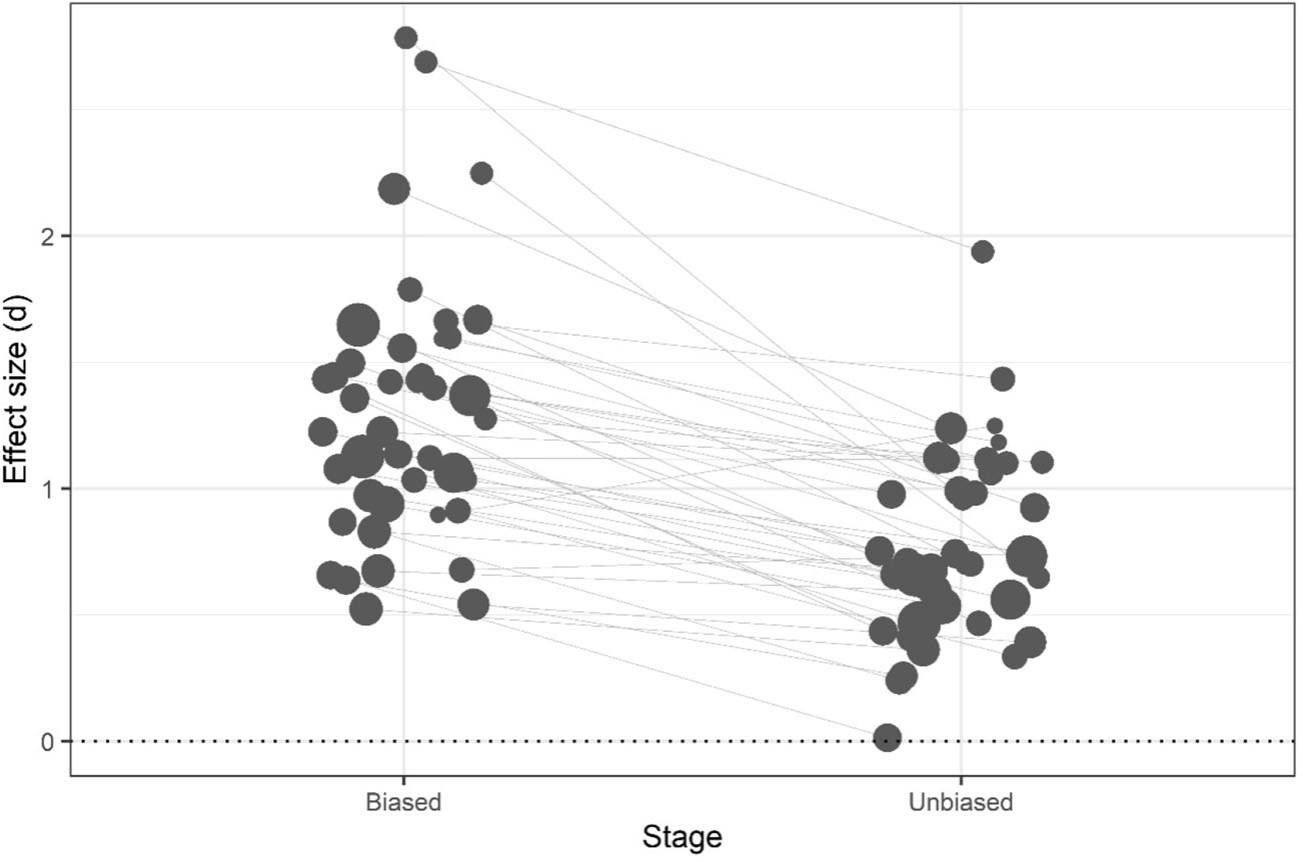
Comparison of effect sizes for the biased and unbiased stages of each study. Effect size (*d*_z_) of the probabilistic cuing effect in visual search in the biased (where targets appear more frequently in one quadrant of the display) and unbiased (where targets appear evenly distributed across the display) stages of each of the studies included in the meta-analysis. The size of each point indicates the sample size of the study

The average size of the probabilistic cuing effect (i.e., the difference in performance between the rich and sparse regions) for the biased stage across all studies (*k* = 42) was large, *d*_*z*_ = 1.19, 95% CI [1.07, 1.32], *z* = 18.16, *p* < .001. For this stage, the meta-analysis also revealed considerable heterogeneity across studies, *Q*(41) = 102.71, *p* < .001, *I*^*2*^ = 59.69%. The average effect size in the unbiased stage was numerically lower, *d*_*z*_ = 0.69, 95% CI [0.60, 0.78], *z* = 14.91, *p* < .001, and heterogeneity was slightly smaller but still significant, *Q*(41) = 70.35, *p* = .003, *I*^*2*^ = 40.42%. A multi-level meta-analysis combining data from both stages and including stage (biased vs. unbiased) as a moderator found that the average effect size was significantly smaller for the unbiased than for the biased stage, *b*_1_ = -0.49, 95% CI [-0.59, -0.39], *z* = -9.44, *p* < .001. Figure 2 shows the effect size for each study during the biased and unbiased stages. Figure S1 in the Online Supplementary Material shows the forest plots for the biased and unbiased stages.

### Moderator analyses

#### Number of trials in the biased and unbiased stages

As explained above, one of the factors that could explain the apparent persistence of the bias is that the unbiased stage usually comprises relatively few trials, too few perhaps to reveal clear evidence of unlearning. A *t*-test confirmed that, as expected, the number of trials in the biased stage (*M* = 377.43, *SD* = 144.75) was significantly larger than in the unbiased stage (*M* = 202.00, *SD* = 128.26), *t*(41) = 7.01, *p* < .001. To test whether the number of trials moderates the results, we carried out separate meta-analyses for each stage adding the number of trials as a moderator. The number of trials did not significantly affect the effect size for either the biased or the unbiased stage, although as expected, the slope of the meta-regression was positive for the biased stage, *b*_1_ = .0002, 95% CI [-.0007, .0012], *p* = .63, and negative for the unbiased stage, *b*_1_ = -.0004, 95% CI [-.0011, .0003], *p* = .25. Figure S2 in the Online Supplementary Material shows the relationship between the number of trials and the effect size of each study.

#### Change in experimental setting

To test whether the difference between stages was mainly driven by studies in which the experimental setting changed between the biased and unbiased stages, we repeated the multi-level meta-analysis but including only those studies where nothing changed from one stage to the next and the transition was not marked in any manner (*k* = 25). This analysis replicated the results of the meta-analysis including all the studies: The average effect size in the unbiased stage was statistically smaller than in the biased stage, *b*_1_ = -0.48, 95% CI [-0.60, -0.36], *z* = -7.65, *p* < .001. Figure S3 in the Online Supplementary Material shows the effect sizes for the biased and unbiased stages separately for studies in which the transition between stages was perceptible and those in which it was not.

### Analyses of publication bias

**Fig. 3:**
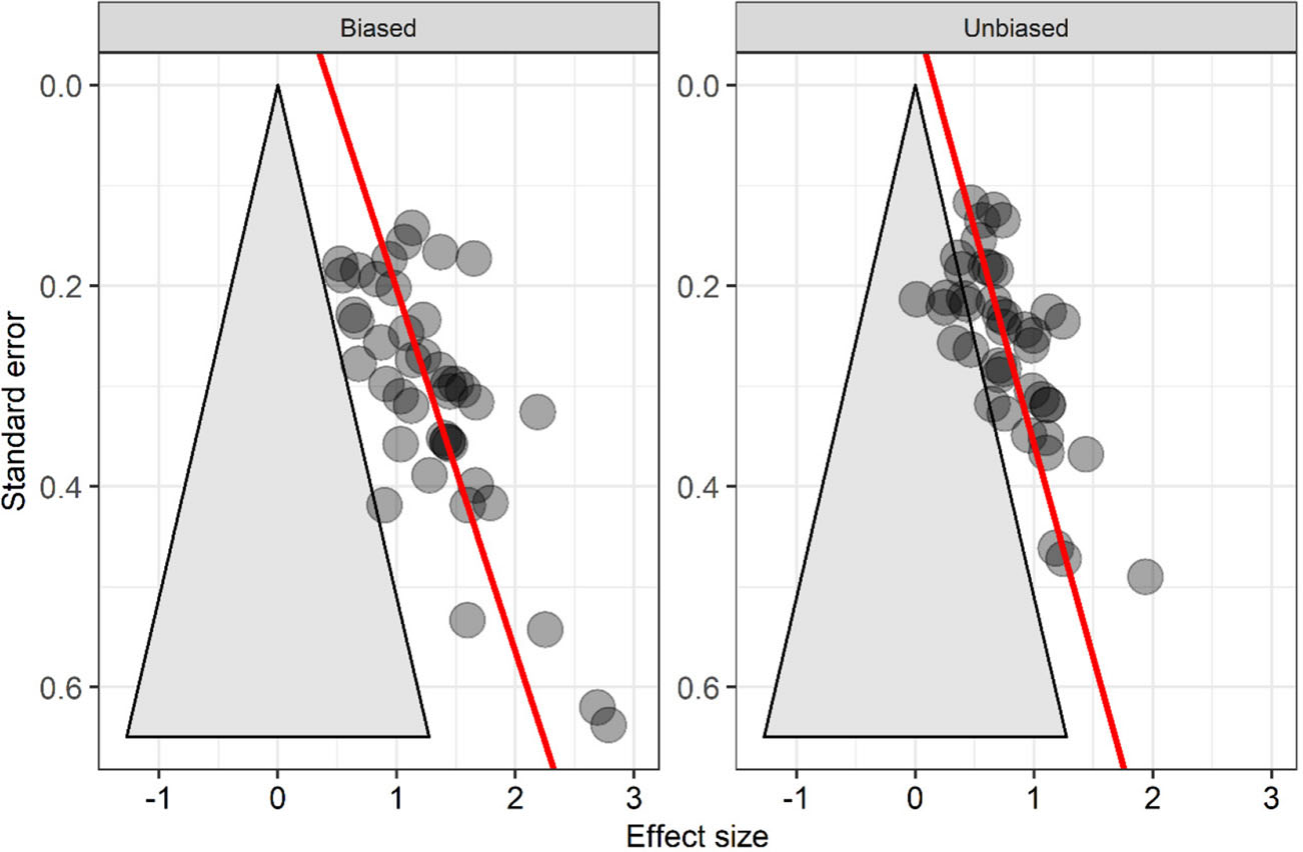
Funnel plots for the biased and unbiased stages of the studies included in the meta-analysis. Effect size plotted against the standard error for the biased and unbiased stages. The red line represents the best-fitting meta-regression of effect sizes on standard errors (Egger’s regression test). The triangle defines the approximate area where effect sizes are non-significant in a two-tailed*t*-test for repeated measures with alpha = .05

Figure 3 shows funnel plots for the effect sizes in both stages. Egger’s regression test (Egger et al., 1997) showed that the distribution of effect sizes was asymmetric for both the biased, *b*_1_= 2.77, 95% CI [1.59, 3.95], *p* < .001, and unbiased stages, *b*_1_ = 2.35, 95% CI [1.28, 3.41], *p* < .001. Table 1 shows the bias-corrected effect sizes returned by PET, PEESE, trim-and-fill, and the selection model separately for each stage. All methods suggest that probabilistic cuing remains significant in both stages after correcting for bias (except for the unbiased stage with the PET method). In all cases, the effect sizes for the unbiased stage remain smaller than for the biased stage.

**Table 1:** Publication bias correction

Method	Biased stage	Unbiased stage
Random effects	1.19 [1.07, 1.32]	0.69 [0.60, 0.78]
PET	0.44 [0.10, 0.77]	0.16 [-0.09, 0.41]
PEESE	0.83 [0.65, 1.00]	0.42 [0.29, 0.55]
Trim-and-fill	1.02 [0.87, 1.16]	0.58 [0.48, 0.68]
Selection model	1.07 [0.95, 1.19]	0.57 [0.41, 0.72]

*Note*. Meta-analytic effect size and 95% confidence interval for each stage and bias-correction method

## Discussion

A meta-analysis of 42 studies confirmed that probabilistic cuing is significantly reduced from the biased to the unbiased stage. Overall, probabilistic cuing during the unbiased stage was roughly half the size of cuing during the biased stage. In our previous empirical study on this issue (Giménez-Fernández et al., 2020), the standard deviation of the probabilistic cuing effect (in the Unbiased-first groups from Experiments 1 and 2) in both stages was approximately 115 ms. Taking this estimate as a yardstick, the average size of the meta-analytic probabilistic cuing effect during the biased stage, *d*_z_ = 1.19, translates to a search advantage of 136 ms for targets in the rich region, while the effect size for the unbiased stage, *d*_z_ = 0.69, corresponds to a search advantage of 79 ms. The decrease in the size of probabilistic cuing occurred when taking into account only studies without any perceptible change between stages. Additionally, the decrease was evident even after correcting for publication bias using several methods.

This is the first meta-analytic evidence that the bias in probabilistic cuing is effectively reduced when the spatial distribution of the target becomes unbiased. These results are consistent with some individual studies reporting the same pattern (e.g., Chua & Gauthier, 2016; Giménez-Fernández et al., 2020; Jiang & Won, 2015). We hypothesized that one of the reasons why some empirical studies might have failed to detect a decrease in probabilistic cuing during the unbiased stage is that most experiments do not include enough unbiased trials. Although most studies include a relatively long biased learning stage, few studies include more than 200 trials in the unbiased stage. Thus, it is difficult to know whether the bias would diminish after sufficient experience with the unbiased spatial distribution of the target. Although we found that the average number of trials was significantly lower in the unbiased than in the biased stage, the number of trials did not significantly moderate the magnitude of probabilistic cuing during the unbiased stage. This non-significant result must be interpreted with caution, though, as among the studies published so far there is little variability in the number of trials in the unbiased stage.

It could be argued that our results actually confirm that probabilistic cuing is an inflexible attentional bias, since the effect remained significant during the unbiased stage and this is unequivocal evidence that the bias persists. Indeed, one of the findings that has been put forward to defend the claim that probabilistic cuing is an inflexible process is that it is still present during the unbiased stage (Jiang, 2018; Jiang & Sisk, 2019; Seger, 2018). That is, response times remain faster when the target appears at the previously rich quadrant than when it appears at the other quadrants, even with this attentional bias is no longer advantageous. However, it is important to note that some persistence of the bias during the unbiased stage makes perfect sense if a viewer is employing goal-driven attention voluntarily: Even in studies where both stages are of the same length, once the unbiased stage is finished, it is undeniably true that the target has appeared more frequently in the rich quadrant. Given that participants are not instructed explicitly about the new spatial distribution of the target (i.e., they have to discover it), and that the cost of maintaining the bias during the unbiased stage is relatively low, it is perfectly reasonable to maintain the bias from a goal-driven point of view. In other words, the persistence of the attentional bias does not necessarily imply that it is driven by an automatic and inflexible habit.

Ultimately, whether or not probabilistic cuing qualifies as an attention habit depends on what is meant by a habit – a longstanding matter of debate and controversy itself (De Houwer, 2019; Luque & Molinero, 2021). One of the defining features of habits is that they are independent from goals and, thus, insensitive to changes in the value or the contingency of rewards (Dickinson, 1985; Wood & Rünger, 2016). Sometimes, in contrast with this definition, in the attention field, authors seem to adopt a “weak” definition of habit, by which an “attentional habit” would be a bias produced by experience that lasts longer than a priming effect. For instance, Jiang and Sisk (2019) state that “researchers have begun to use the term ‘habit’ to describe enduring attentional biases, contrasting them with short-term changes such as inter-trial repetition priming” (p. 65). If the term is used, as in these cases, as a simile or heuristic, without implying that the same mechanisms underlie the habits studied in reward-learning protocols and attentional habits such as probabilistic cuing, then our results are consistent with the conclusion that probabilistic cuing is a habit, because it does remain significant over the unbiased stage. However, if we adopt a “strong” use of the term “attentional habit” (see Anderson, 2016; Jiang & Sisk, 2019), then we believe that there is insufficient evidence for claiming that probabilistic cuing matches this definition. On this conception, attentional biases are similar to habits observed in the reward learning literature: these are behaviors acquired through reward learning that are maintained fixed despite change in their consequences, even across hundreds of trials (e.g., Bouton, 2019; Hardwick et al., 2019).

Beyond the inflexibility argument, it has been claimed that probabilistic cuing shares other features with habits: that it is egocentric (Jiang, Swallow, & Sun, 2014a), robust to increases in working memory load (Won & Jiang, 2015), exhibits task-specificity (Addleman et al., 2018), and is unconscious (Jiang, Sha, & Sisk, 2018). All these questions deserve further study, but we would like to point out that at least the argument that probabilistic cuing is unconscious has also been challenged. Contrary to the popular view that probabilistic cuing is an unconscious learning effect, meta-analyses and high-powered empirical studies (Giménez-Fernández et al., 2020; Vadillo et al., 2020) reveal that (a) participants consciously detect the biased distribution of the targets during the initial learning stage, (b) they also consciously detect the change in contingencies during the unbiased stage, and (c) even after the unbiased stage they still know that, over the experiment, the target appeared more frequently in the rich quadrant. Taken collectively, these results suggest that participants’ performance in the visual search task is highly consistent with their conscious knowledge of the spatial distribution of targets. Given that conscious knowledge and performance in the task seem to change in tandem over the course of the experiment, it is premature to discard the possibility that probabilistic cuing is actually a goal-driven attentional bias.

In any case, the theoretical debate about the underlying mechanisms of probabilistic cuing and related phenomena is unlikely to make real progress unless future studies include much larger sample sizes. In this specific case, although many individual studies failed to detect a decline of probabilistic cuing during the unbiased stage, our results suggest that this is most likely due to the lack of statistical power. Crucially, the argument that probabilistic cuing is a habit because it is persistent loses its force if the experiments supporting this claim are designed in such a way that changes in behavior cannot be detected reliably. In general, low statistical power is a recurrent problem in the area of unconscious learning that obscures the interpretation of non-significant results and that needs to be addressed in future research (Vadillo et al., 2016).

In conclusion, our results challenge the claim that probabilistic cuing is inflexible, and suggest instead that participants tend to reduce their bias to the previously rich quadrant as they accumulate more and more evidence that the target is evenly distributed. We propose that, with the data at hand, we cannot discount the possibility that probabilistic cuing is the outcome of a goal-driven attentional process.

## Supplementary Information

ESM 1 (DOCX 865 kb)
