# Supplementary material for: Is probabilistic cuing of visual search an inflexible attentional habit? A meta-analytic review
Source: Psychon Bull Rev. 2021 Nov 23;29(2):521–9. doi: 10.3758/s13423-021-02025-5 (PMC9038896; doi:10.3758/s13423-021-02025-5)
Supplement: Supplementary file 1 — (DOCX 865 kb) [file 13423_2021_2025_MOESM1_ESM.docx]

**SUPPLEMENTARY MATERIAL**

**Is probabilistic cuing of visual search an inflexible attentional habit? A meta-analytic review**

Tamara Giménez-Fernández, David Luque, David R. Shanks, & Miguel A. Vadillo

**Figure S1. Forest plots for the biased and the unbiased stages.** The code for each study refers to the first letter of the surname of each author, the year of publication and an identification of the study number, condition, or sample of participants, if the article contained more than one study. Usually, this identification is numerical, but in some cases, they are alphabetical: In this case A stands for Aware, U for Unaware, P for Parkinson and C for Controls. For each study, the last two columns in each panel show the effect size (Cohen’s d_z_) and its 95% confidence interval.


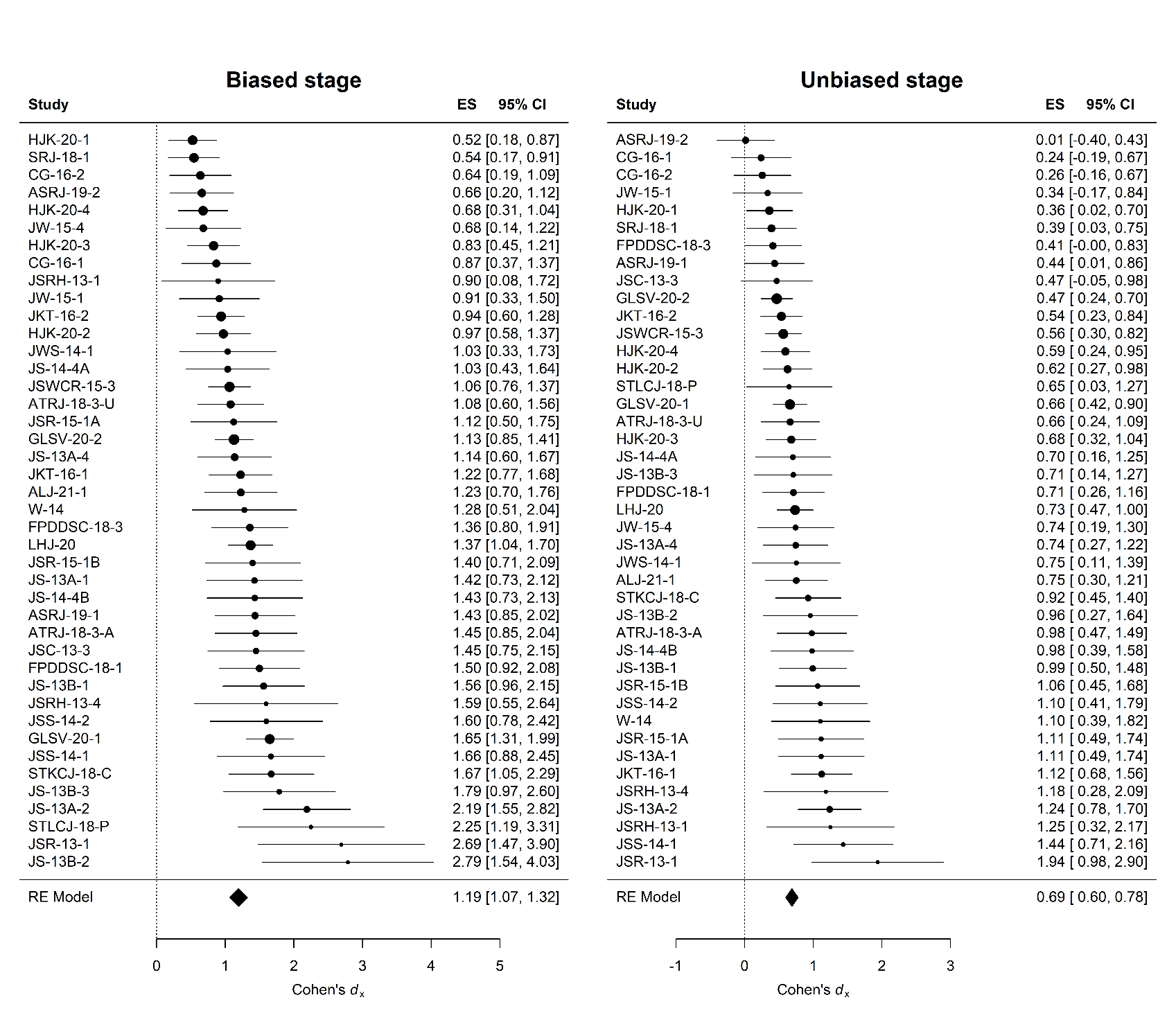


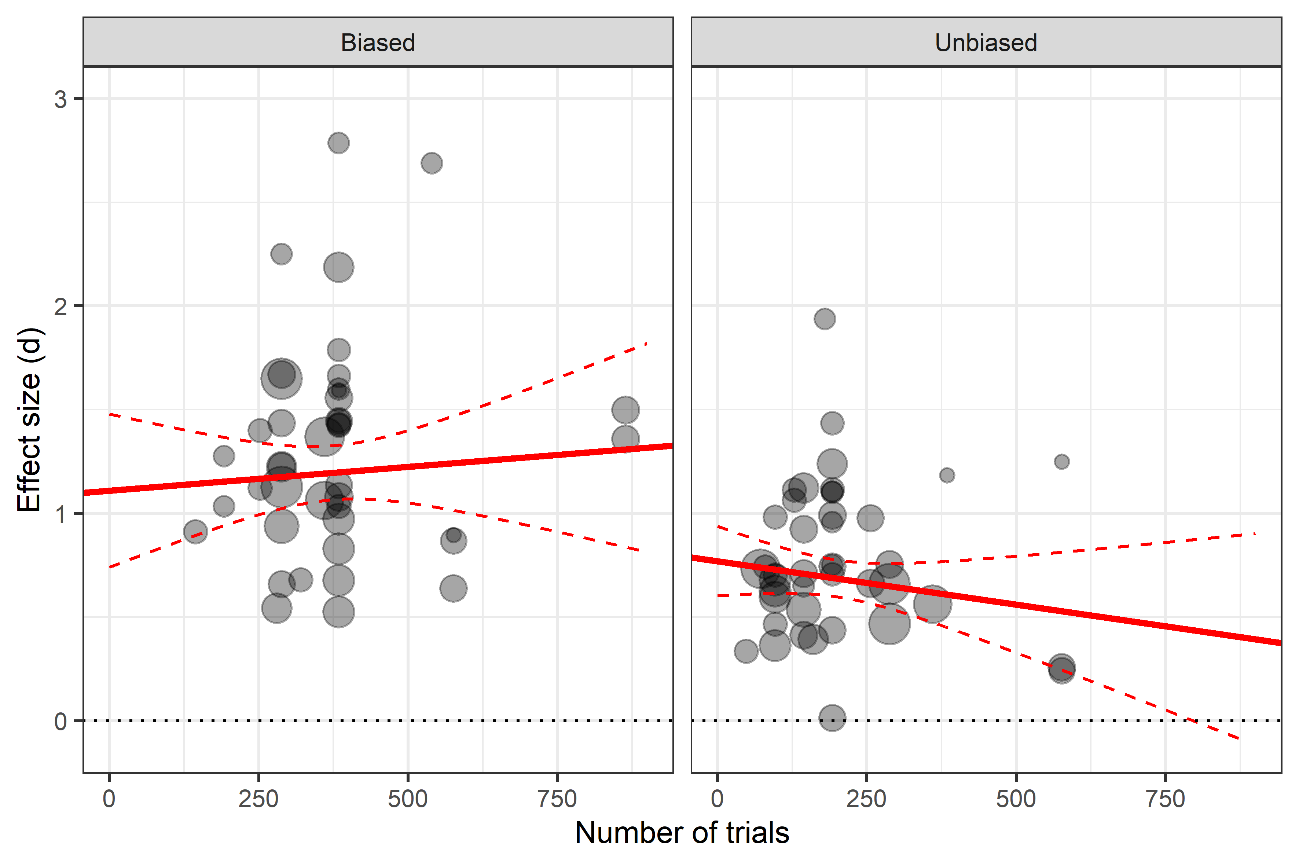


**Figure S2. Scatterplot for the effect size against the number of trials in each stage.** Each point represents one study included in the meta-analysis. The *y* axis refers to the effect size in Cohen’s *d*_z_ units and the *x* axis represents the number of trials included in the stage. The size of each point depends on the study’s sample size. The red line represents the best-fitting meta-regression between the number of trials and the effect size for each stage. The dotted red line represents the 95% confidence interval of the meta-regression.


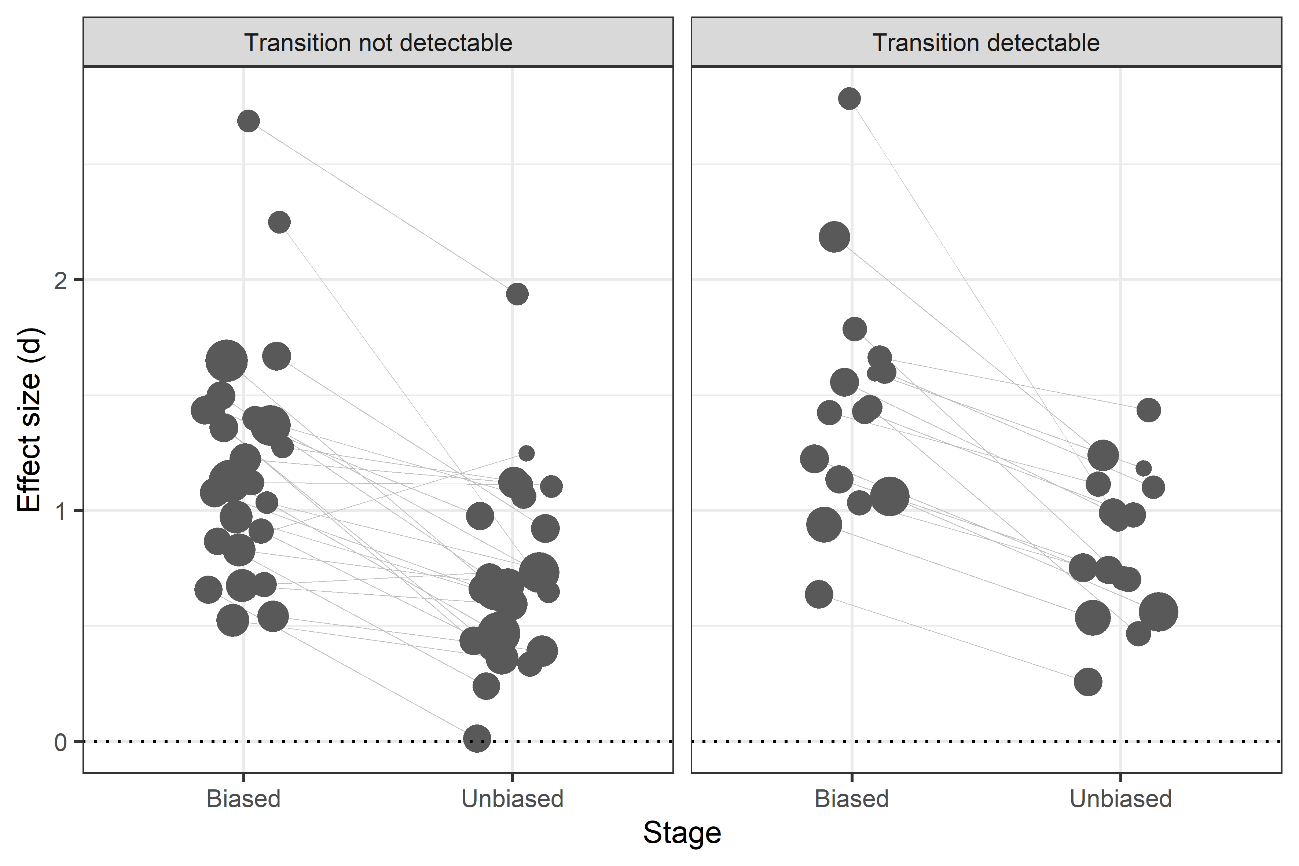


**Figure S3. Comparison of effect sizes for studies in which the transition between stages was perceptible or not for participants**. The left-hand panel shows effect sizes for the biased and unbiased stages in studies in which the transition between stages was not perceptible for participants. The right-hand panel shows the same information for studies in which the transition between stages was perceptible for participants (i.e., something changed in the procedure or there was a time-break between the stages). The size of each point depends on the study’s sample size.
